# Supplementary material for: Five-Year Mortality of Patients with Perioperative Myocardial Infarction After On-Pump Isolated or Combined Coronary Artery Bypass Graft Surgery: A Retrospective Propensity Score-Weighted Analysis
Source: J Clin Med. 2025 Nov 10;14(22):7970. doi: 10.3390/jcm14227970 (PMC12653309; doi:10.3390/jcm14227970)

Supplementary Files

Table S1 – Imbalance of patient's characteristics before and after propensity weighting in assessing 5-year all-cause mortality.

Variables	Before weighting			After weigthing		
**Variables**	**No pMI (n=600)**	**pMI (n=112)**	**SMD**	**No pMI (n=590)**	**pMI (n=85)**	**SMD**
Age (years)	68 ± 9	67 ± 11	0.129	68 ± 9	68 ± 10	0.011
Logistic EuroSCORE 2 (%)	5.3 ± 3	6.5 ± 4	0.301	5.5 ± 3	5.3 ± 3	0.024
Preoperative Hemoglobin (g/dl)	13.3 ± 1.8	13.1 ± 1.9	0.113	13. 3 ± 1.7	13.4 ±1.7	0.005
Preoperative Glomerular filtration rate (ml/min/1.73 m^2)	80 ± 27	77 ± 29	0.120	79 ± 28	80 ± 27	0.027
Acute Heart Failure before surgery	53 (9)	16 (14)	0.171	58 (10)	11 (10)	0.020
NSTEMI	121 (20)	28 (25)	0.249	124 (21)	24 (21)	0.004
Administration of RBC during surgery	103 (17)	31 (28)	0.254	112 (19)	20 (18)	0.020
Redo surgery for bleeding	28 (5)	15 (13)	0.301	36 (6)	7 (6)	0.006

Data are presented as mean ± standard deviation or as numbers (percentages).

An absolute MSD < 10% supported the assumption of a group balance**.**

NSTEMI: non-ST-elevation myocardial infarction; **RBC**: red blood cell; **SMD**: standardized mean differences

Figure S1 – Five-Year Survival Curve of Isolated CABG Patients

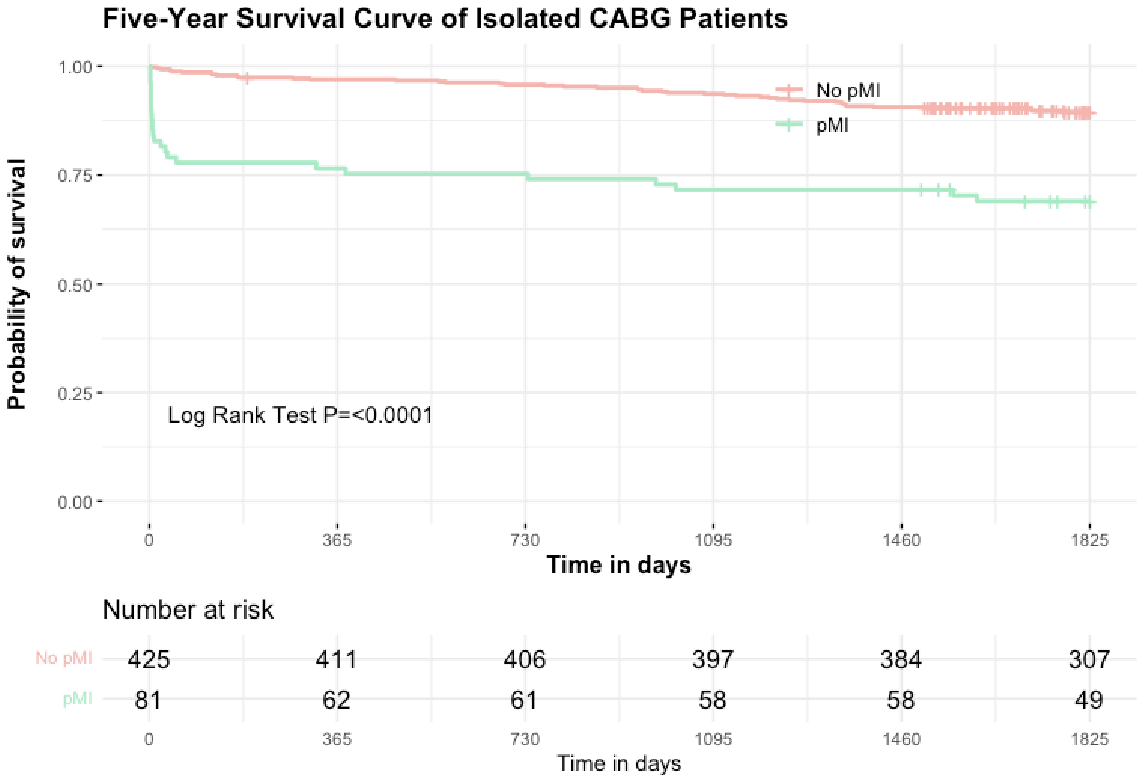

Figure S2 - Landmark analysis of 5-year survival starting 30 days postoperatively.

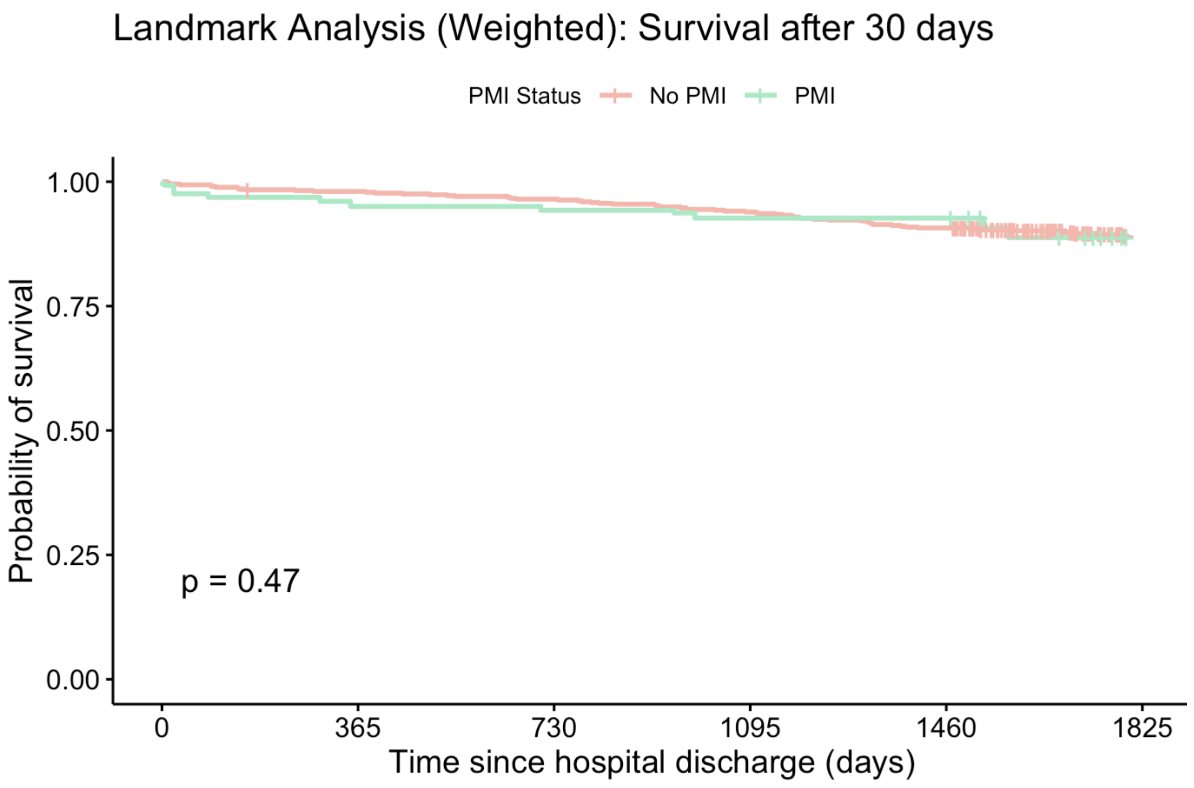

Supplement: Supplementary file 1 [file jcm-14-07970-s001.zip › jcm-3914894-supplementary.pdf]
